# A Rare Presentation of Alveolar Rhabdomyosarcoma of the Sinonasal and Nasopharyngeal Region With Bone Marrow Metastasis

**DOI:** 10.7759/cureus.107812

**Published:** 2026-04-27

**Authors:** Mohamed Al Tatari, Juliet Ramirez, Stephanie Hernandez, Ever Martinez, Damian Casadesus

**Affiliations:** 1 Internal Medicine, Jackson Memorial Hospital, Miami, USA; 2 Medicine, American University of the Caribbean School of Medicine, Cupecoy, MAF; 3 Medicine, Ross University School of Medicine, Bridgetown, BRB

**Keywords:** adult soft tissue sarcoma, alveolar rhabdomyosarcoma, bone marrow metastasis, nasopharyngeal carcinoma, sinonasal tumor

## Abstract

We report the case of a Hispanic woman in her 50s who presented to the emergency department with persistent hip and left lower extremity pain, followed by progressive anosmia and a right-sided neck mass. On physical examination, the patient had a palpable lymph node in the right submandibular area, and she reported a history of anosmia. A CT scan of the neck and face revealed an enhancing mass in the superior nasal cavity/ethmoid sinus with cribriform plate dehiscence and pachymeningeal enhancement and a right cervical lymph node. A biopsy of the lymph node revealed alveolar rhabdomyosarcoma (ARMS) from the sinonasal and nasopharyngeal region. Further studies revealed bone marrow metastatic disease. The patient started chemotherapy with vincristine, doxorubicin, and ifosfamide. Rhabdomyosarcoma is the most common soft tissue sarcoma in the pediatric population, but it is rare in adults. Moreover, the ARMS subtype, which is also found in the nasal cavities, is even less common in adults. Rhabdomyosarcoma is typically seen in the pediatric population as a rare soft tissue tumor, but it is even more uncommon in adults. Current treatment protocols in adult ARMS are extrapolated from pediatric regimens, but adults often experience greater toxicity and have poorer outcomes, highlighting the need for adult-specific clinical trials. This case is a great example of the importance of timely recognition, comprehensive diagnostic workup, and multidisciplinary management approaches for the optimal care of rare adult ARMS.

## Introduction

Alveolar rhabdomyosarcoma (ARMS) is a high-grade soft tissue sarcoma derived from primitive mesenchymal cells with skeletal muscle differentiation, characterized by PAX3-FOXO1 or PAX7-FOXO1 gene fusions in most cases. It is classically a pediatric malignancy with aggressive local behavior and a propensity for early metastasis to the lungs, bone, and bone marrow. Early detection, histopathologic confirmation, and multimodal treatment underpin improved outcomes in children [1,2].

Although rhabdomyosarcoma (RMS) is relatively uncommon even in the pediatric population, its occurrence in adults is rare and carries a worse prognosis [3,4]. Adult presentations are heterogeneous, and because prospective data are limited, therapeutic decision-making frequently relies on extrapolating pediatric treatment paradigms; and this tension between pediatric-derived regimens and adult disease biology remains a subject of ongoing debate [3-6]. Head and neck involvement in older adults, particularly the alveolar subtype, adds diagnostic complexity because of atypical immunoprofiles and the challenge of distinguishing these lesions from other small round cell neoplasms without molecular confirmation [2]. The difficulty of securing a definitive diagnosis in rare tumors is well-illustrated by a recent case, where classic radiological features failed to yield histopathological confirmation despite multiple examinations [7]. This reinforces the necessity of pursuing molecular studies when presentations are atypical.

This case report documents a patient with a rare presentation because it involves ARMS in a middle-aged adult with a primary tumor in the sinonasal and nasopharyngeal region, locations far less commonly reported in adults than in children, alongside early and extensive metastatic spread, including bone marrow infiltration and a dural-based intracranial lesion. 

## Case presentation

A Hispanic woman in her 50s with a medical history of hypertension, pre-diabetes, iron-deficiency anemia, and prolactinoma treated with cabergoline presented to the emergency department with two weeks of intractable bilateral lower extremity pain in the setting of one month of progressive anosmia, ageusia, nasal congestion, and a right-sided cervical mass. She denied headaches, rhinorrhea, vision changes, or dysphagia, but endorsed fatigue, weight loss, and progressive leg discomfort. In the emergency department, she was hemodynamically stable and afebrile. Upon physical examination, cardiovascular, pulmonary, and abdominal examinations were normal. Neck examination revealed a 5-centimeter mobile painless mass on the right side of the neck. In the neurological examination, the patient complained of anosmia. Initial laboratory evaluations are reported in Table 1.

**Table 1:** Key laboratory trends ^†^Values inferred from partially truncated "fishbone" entries. Hyphens (-) indicate not reported. WBC: white blood cell; ANC: absolute neutrophil count; Hgb: hemoglobin; MCV: mean corpuscular volume; Ca: calcium; Cr: creatinine; AST: aspartate aminotransferase; ALT: alanine aminotransferase; CRP: C-reactive protein

Date (2025)	Clinical context	WBC (×10⁹/L)	ANC (×10⁹/L)	Hgb (g/dL)	Platelets (×10⁹/L)	MCV (fL)	Ca (mg/dL)	Cr (mg/dL)	AST/ALT (U/L)	CRP (mg/L)
Day 1	ED/admission baseline	9.8	-	9.8	161	76.5	10.4	0.73	61/20	13.8
Day 9	Pre-treatment	8.8	-	~9.3^†^	148	-	-	0.70	-	-
Day 16	Pre-treatment (progression)	~5.3^†^	-	9.3^†^	88	-	-	-	-	-
Days 30-32	Post-VAI C1D5 nadir	0.7	0.2	9.7	34	-	-	-	-	-
Reference ranges		4.5-11.0 × 10^9^/L	1.5-8.0 × 10⁹/L	Female: 12.0-16.0 g/dL	150-400 × 10^9^/L	80-100 fL	8.4-10.2 mg/dL	0.6-1.2 mg/dL	AST: 12-38 U/L; ALT: 10-40 U/L	>9 mg/L

**Figure 1:**
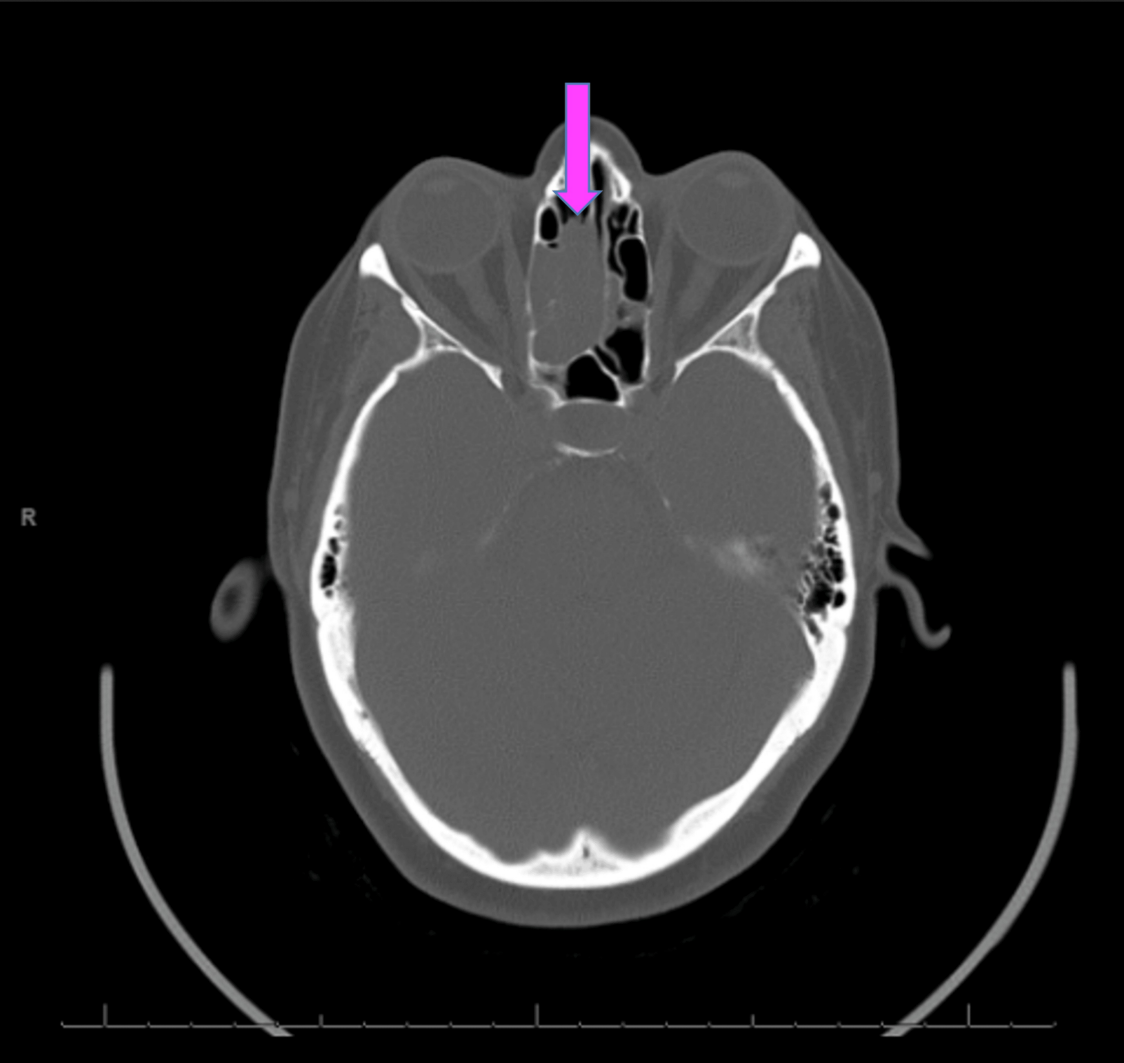
CT scan of the brain revealed a 3.1 × 2.0 × 1.4 centimeter enhancing mass in the superior nasal cavity

A flexible nasopharyngolaryngoscopy revealed a red mass in the superior nasal cavity near the cribriform plate region, without purulence or drainage. A CT scan of the brain without contrast revealed an incidental partially calcified, dural-based lesion along the right parietal convexity with hyperostosis, consistent with a meningioma and unrelated to the patient's presenting symptoms. It also revealed a 3.1 × 2.0 × 1.4 centimeter enhancing mass in the superior nasal cavity and ethmoid sinus with cribriform plate dehiscence (Figure 1).

**Figure 2:**
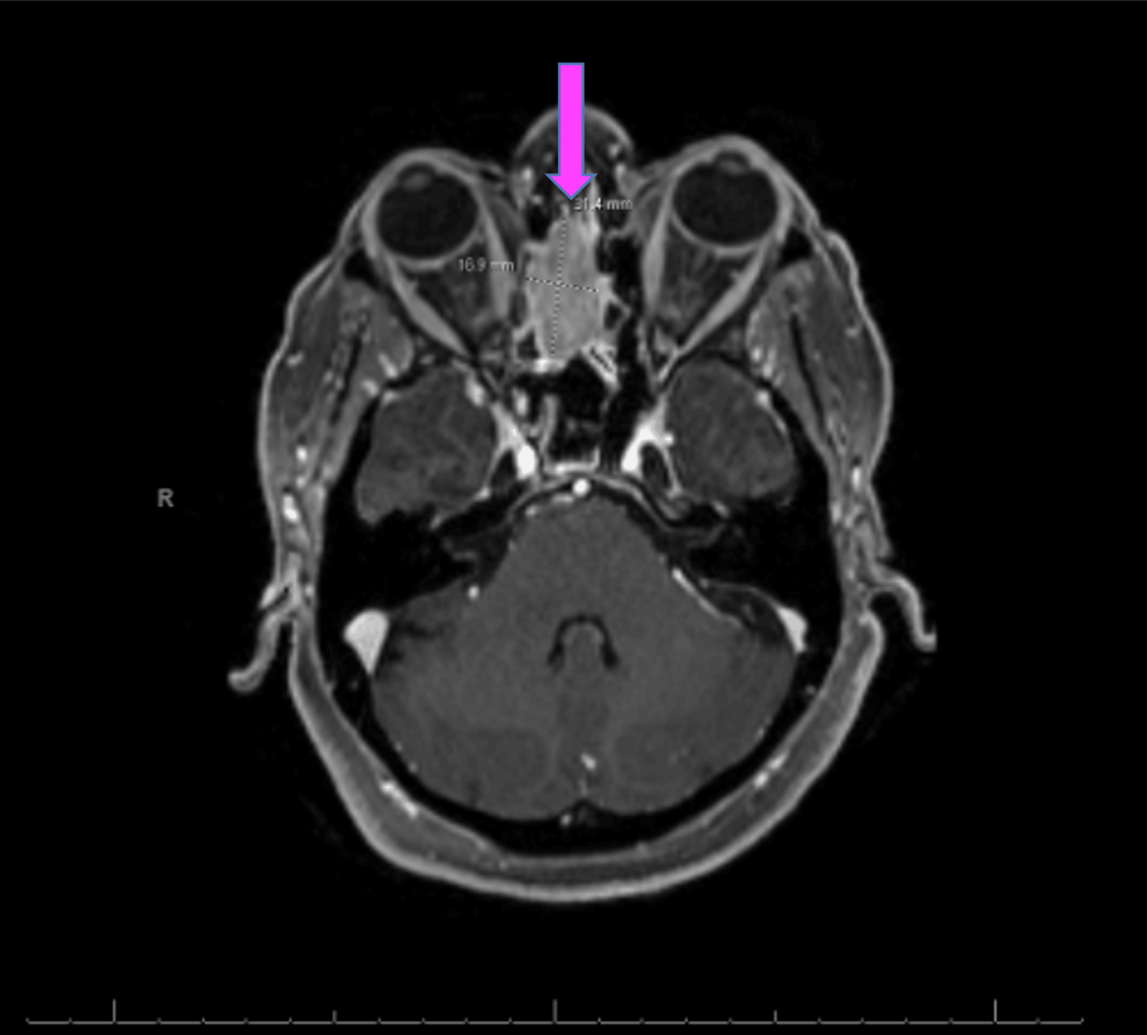
MRI of the brain confirmed the upper nasal cavity mass

**Figure 3:**
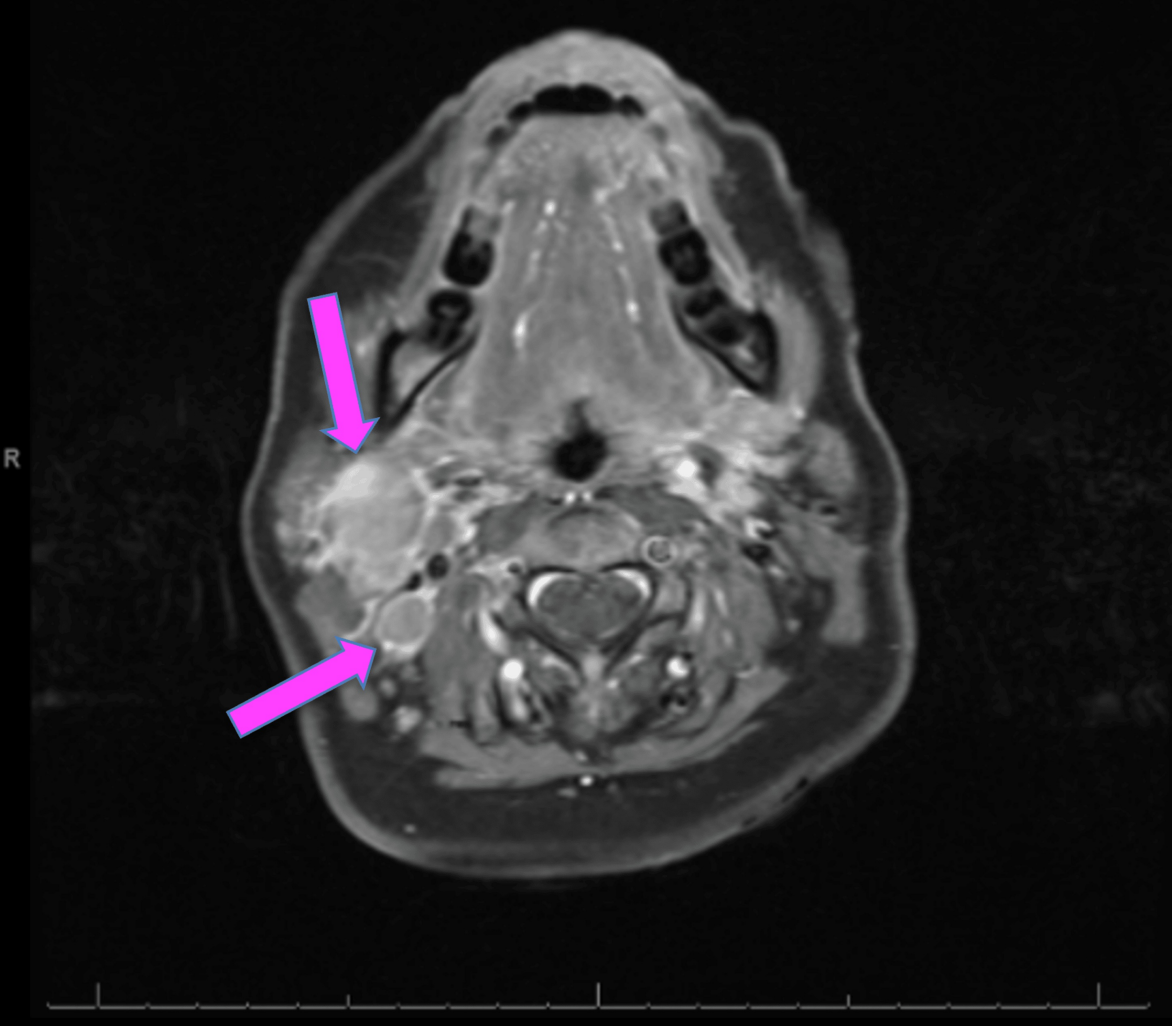
MRI of the neck revealed multiple right side lymph nodes, the largest being 2.3 cm

**Figure 4:**
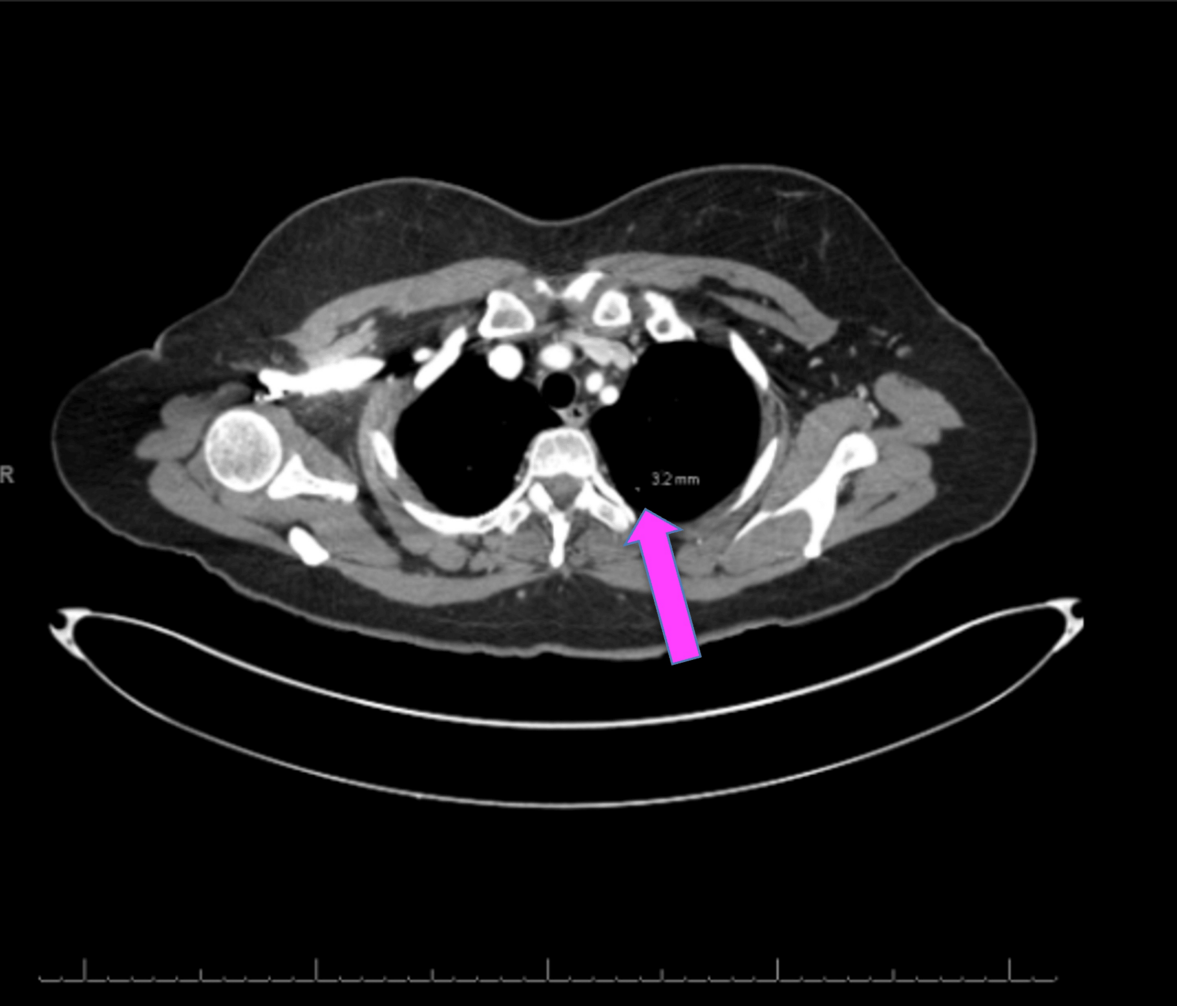
CT scan of the chest revealed left side pulmonary nodule

**Figure 5:**
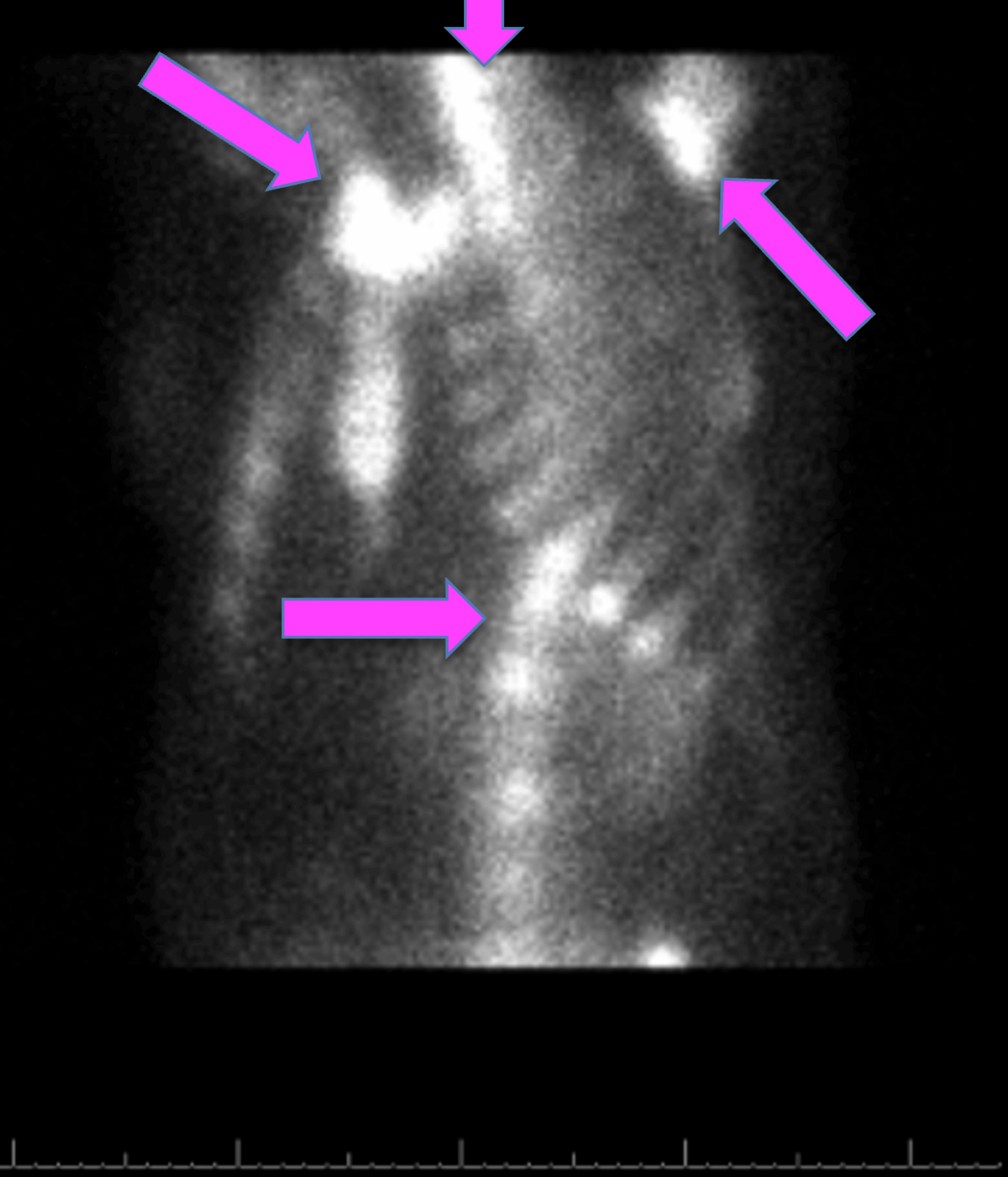
Widespread bone metastasis was observed in the nuclear medicine bone scan

MRI of the brain confirmed the mass described in the CT scan of the brain (Figure 2). MRI of the neck revealed multiple right-sided lymph nodes, the largest measuring 2.3 cm, causing extrinsic compression and narrowing of the right internal jugular vein. No thrombus was identified, and the patient remained asymptomatic from a vascular standpoint (Figure 3). A CT scan of the chest revealed multiple pulmonary nodules measuring 3.2 mm (Figure 4). A whole-body bone scintigraphy was performed and revealed widespread osseous metastases involving the bilateral humeral and femoral bones, pelvis, and spine (Figure 5). MRI of the lumbar spine further revealed diffuse marrow abnormalities with chronic compression deformities but no cord compression.

**Figure 6:**
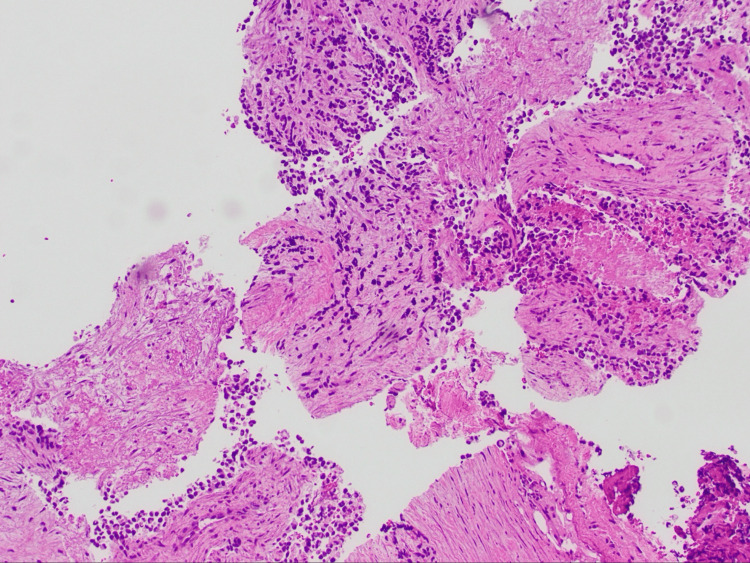
Rhabdomyosarcoma (H&E: 10×) H&E: hematoxylin and eosin

**Figure 7:**
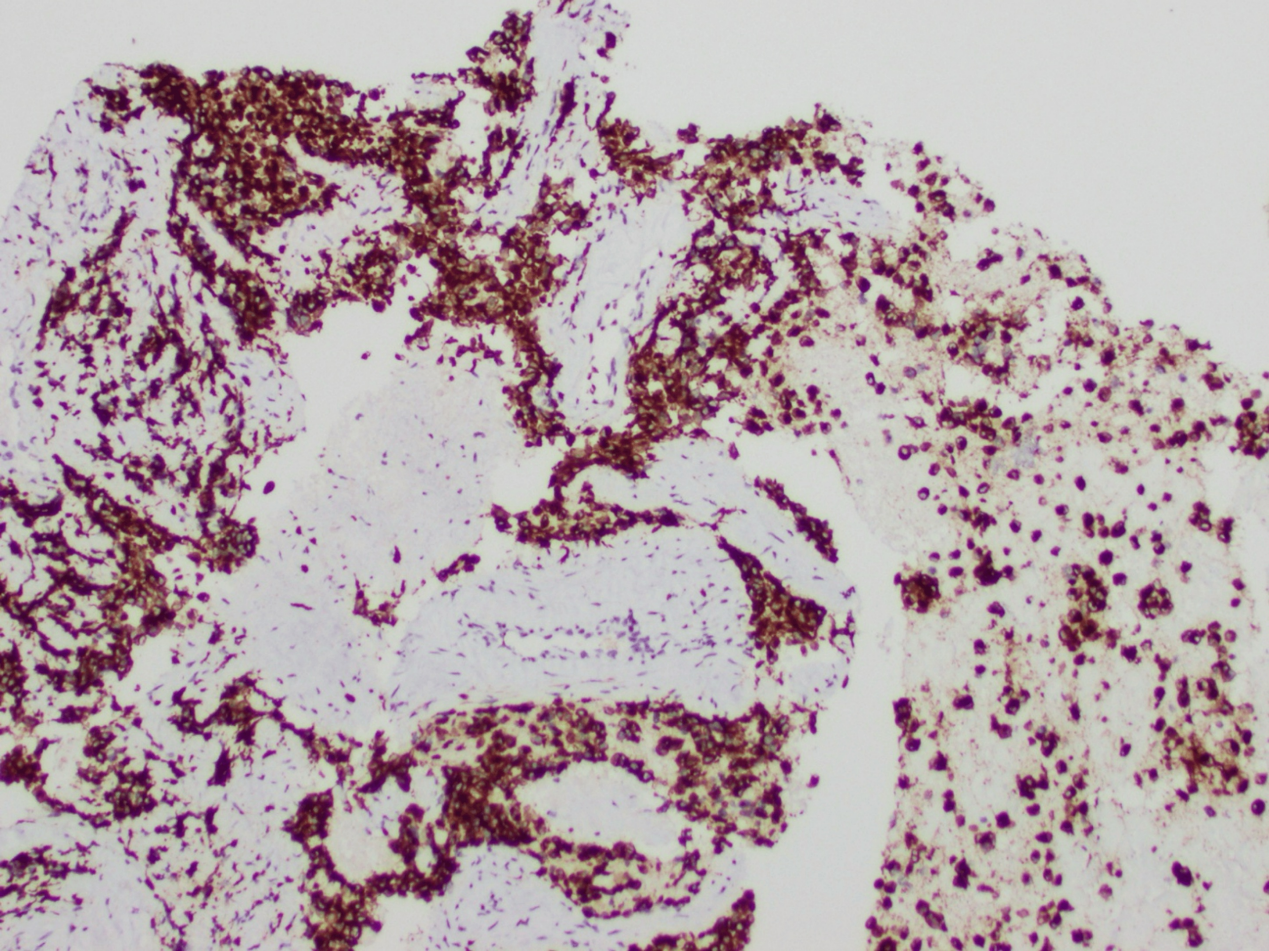
Desmin staining of rhabdomyosarcoma (10×)

**Figure 8:**
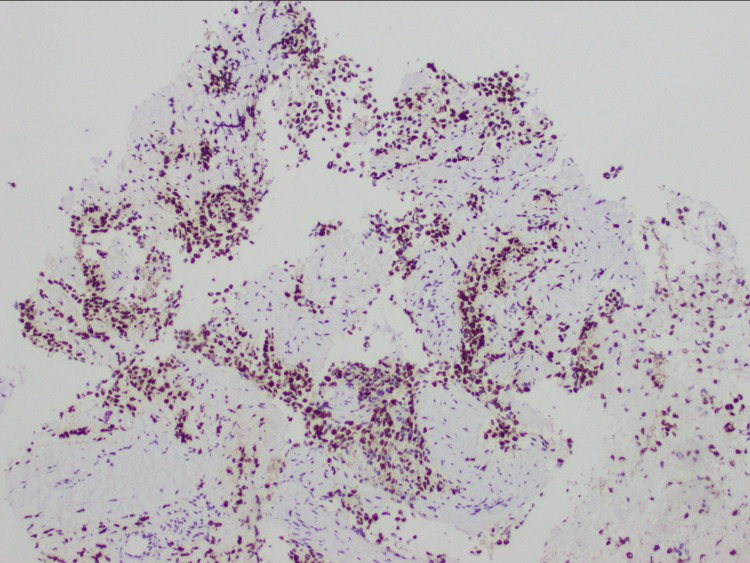
MGN staining of rhabdomyosarcoma (10×) MGN: myogenin

**Figure 9:**
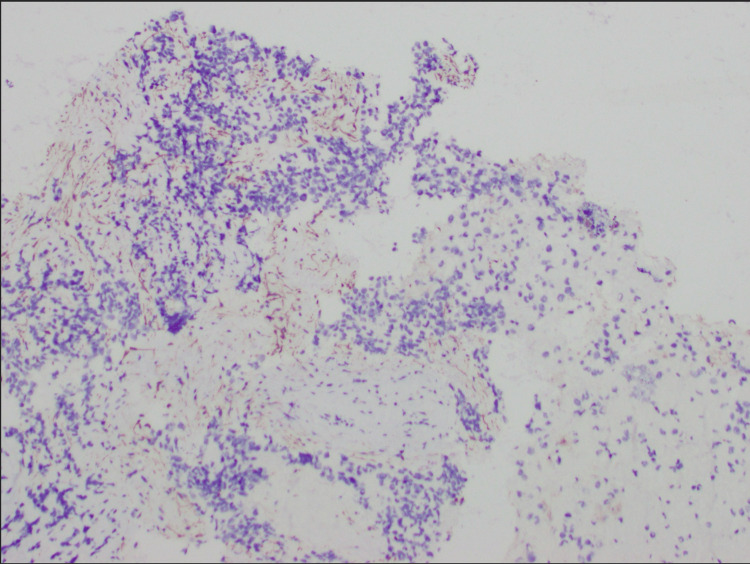
AE13 staining of rhabdomyosarcoma (10×)

A biopsy of the cervical lymph node was performed and revealed high-grade ARMS (Figure 6), positive for desmin (Figure 7), myogenin (Figure 8), and AE13 (Figure 9) and negative for epithelial and neuroendocrine markers.

She initiated first-line chemotherapy with vincristine, doxorubicin, and ifosfamide (VAI regimen) but developed ifosfamide-associated neurotoxicity on cycle 1 day 2, pushing to a temporary discontinuation. She received supportive care including intravenous fluids and high-dose thiamine, though evidence for this approach is limited; methylene blue is the standard treatment but was not administered. Ifosfamide was completed at a slower infusion rate. At discharge, the patient's leg pain had improved with multimodal analgesia, and the neck mass had slightly decreased in size. She was discharged in stable condition with plans to continue systemic therapy as an outpatient.

## Discussion

RMS accounts for 50% of all soft tissue sarcomas in children and is the most common pediatric soft tissue sarcoma [8]. However, they are rare since they comprise only 3-4% of pediatric cancers overall. Osteosarcoma, a primary bone tumor, is more common overall among childhood sarcomas but belongs to a different category. ARMS primarily affects children and adolescents with a bimodal distribution with peaks between the ages of 2-6 and 10-18 years old with a slight male predominance [9]. RMS most commonly affects the head and neck areas (29.4-40%), the urogenital organs (20-25%), and the extremities (18-26.6%) [10,11]. Adult cases of RMS are extremely rare; soft tissue sarcomas comprise less than 1% of all solid tumor malignancies with RMS comprising 3% of all soft tissue sarcomas in adults [12]. In comparison to children, ARMS in adults carries a poorer prognosis. Five-year survival rates vary widely by stage: approximately 60% for localized disease but less than 30% for metastatic disease at presentation [9,12]. There has also been an association with RMS and several genetic syndromes such as neurofibromatosis, Li-Fraumeni syndrome, Beckwith-Wiedemann syndrome, DICER1 syndrome, and Costello syndrome [8].

There are four main subtypes of RMS as classified by the World Health Organization (WHO): embryonal, alveolar, pleomorphic, and undifferentiated. The alveolar subtype comprises approximately 21% of all RMS cases and has a strong tendency to metastasize to distant locations, therefore leading to a poorer prognosis. Less than 25% of patients have metastasis at the time of diagnosis, and the most common areas of spread include the lungs (39%), bone marrow (32%), lymph nodes (30%), and bones (27%) [11,13]. Diagnosis of ARMS requires histopathology, immunohistochemistry, and molecular confirmation. The hallmark translocations t(2;13)(q35;q14) and t(1;13)(p36;q14), resulting in PAX3-FOXO1 and PAX7-FOXO1 fusions, respectively, are associated with adverse outcomes and refine prognostic assessment [13].

Relevant literature on adult sinonasal/nasopharyngeal ARMS is sparse. Pediatric series emphasize these para-meningeal sites as high-risk and diagnostically challenging, underscoring the need for vigilance when non-specific sinonasal symptoms occur [6]. Adult head and neck ARMS case series and reviews highlight worse outcomes compared to pediatric patients, the difficulty in establishing consensus treatment guidelines, and the common practice of adapting pediatric multimodal protocols for adults [2,4,5]. Case reports of adult sinonasal or nasopharyngeal ARMS, including both alveolar and embryonal variants, demonstrate variability in presentation and generally aggressive courses, with isolated reports of unusual metastatic patterns such as breast involvement or early spinal dissemination [10,11].

In adults, the pleomorphic subtype is more common, making this patient's presentation with ARMS at age 51 particularly rare [12]. The unfavorable primary site in the ethmoid and superior nasal cavity with cribriform plate extension further worsened the prognosis due to the risk of intracranial spread [6]. Widespread osseous and marrow involvement at diagnosis is uncommon but has been reported in comparable adult cases (Table 2) [11,13-16]. The patient presented with mild, asymptomatic hypercalcemia (10.4 mg/dL) without elevated parathyroid hormone, possibly representing a paraneoplastic phenomenon rarely described in ARMS [17]. Calcium normalized without specific intervention.

**Table 2:** Comparison of published cases of adult ARMS of nasal/sinus region with bone marrow metastases CPA: cyclophosphamide; VCR: vincristine; DXR: doxorubicin; DTIC: dacarbazine; IFM: ifosfamide; ETP: etoposide; DACT: dactinomycin

Author/year	Age	Sex	Location	Treatment	Outcome
Bahn and Lee, 2011 [14]	68	F	Sinonasal region with lymph node metastases	4 cycles of CPA + VCR + DXR + DTIC; 5 cycles of IFM + ETP + VCR	Developed a subdural hematoma and died 13 months after diagnosis
Bahn and Lee, 2011 [14]	48	F	Sinonasal region	4 cycles of IFM + ETP + VCR	Developed breast metastases 6 months after diagnosis. Developed sepsis and died 14 months after diagnosis
Aida et al., 2015 [11]	29	F	Nasal cavity	Alternating cycles of VCR + DXR + CPA and ETP + IFM, followed by radiation therapy	Developed brain metastases 15 months after diagnosis. Alive with persistent disease
Asghar et al., 2020 [15]	54	M	Nasopharynx extending into the maxillary sinus	2 cycles of VCR + DACT + CPA	Lost to follow-up
Cheng et al., 2022 [16]	67	F	Sinonasal region	VCR + DACT + CPA	Developed hypoxic respiratory failure due to aspiration, transferring to the ICU. The patient requested hospice care and died prior to discharge home
Ahmad Fahmi et al., 2023 [13]	55	F	Nasal cavity	Unknown	Died due to pancytopenia and systemic infection within days of diagnosis
Current case	51	F	Sinonasal and nasopharyngeal region	VCR + DXR + IFM followed by filgrastim	The patient is stable and being followed on an outpatient basis and prescribed pegfilgrastim

ARMS is an aggressive disease which requires a multimodal approach, incorporating surgery, chemotherapy, and radiotherapy [13]. The management of adult ARMS relies on extrapolation from pediatric regimens, as prospective trials in adults are lacking. The most common chemotherapeutic agents used are vincristine, doxorubicin, dactinomycin, ifosfamide, cyclophosphamide, and etoposide, with the gold standard in treating RMS being vincristine, dactinomycin, and cyclophosphamide [13]. On the one hand, when looking at other similar cases that have been treated with similar regimens, the prognosis seems evidently poor. In our case, the patient received an inpatient treatment regimen of vincristine, doxorubicin, ifosfamide, and filgrastim. Following treatment, the patient was stable, and she was later discharged home with pegfilgrastim to be taken on an outpatient basis with further follow-up appointments to be scheduled with the hematology and oncology department.

## Conclusions

This case of adult sinonasal and nasopharyngeal ARMS with bone marrow metastasis highlights the importance of considering rare malignancies in the differential diagnosis of head and neck masses with atypical features. When a head and neck mass presents with atypical neurologic or hematologic clues, keep ARMS as a differential. Prompt and comprehensive evaluation, including advanced imaging, tissue biopsy with immunohistochemistry, and molecular studies, is essential for timely diagnosis and the initiation of therapy. Outcomes in adults are challenging, but speed and coordination are crucial; medical oncology, radiation oncology, otolaryngology, neurosurgery, pathology, radiology, and supportive care must work in tandem. Early recognition and a disciplined, truly multidisciplinary plan give patients the best chance at meaningful control in a disease that rarely offers second chances.
